# Melasma Management: A Comprehensive Review of Treatment Strategies Including BTX‐A

**DOI:** 10.1111/jocd.16669

**Published:** 2024-11-12

**Authors:** Barbara Kania, Margarita Lolis, David Goldberg

**Affiliations:** ^1^ Skin Laser & Surgery Specialists, A Division of Schweiger Dermatology Group New York New York USA; ^2^ Icahn School of Medicine at Mt. Sinai New York New York USA

**Keywords:** botulinum toxin, hyperpigmentation, melasma

## Abstract

**Background:**

Botulinum toxin A (BTX‐A) is a widely utilized protein derived from the bacterium *Clostridium botulinum* , known for its effectiveness in treating various medical conditions involving muscle spasticity, involuntary muscle movements, and pain disorders. Beyond its therapeutic applications, BTX‐A is also commonly used in cosmetic procedures to address dynamic wrinkles, hyperhidrosis, sebum production, pore size, and overall skin texture. While the use of neurotoxins like BTX‐A for treating conditions such as UVB‐induced hyperpigmentation, specifically melasma, is an emerging area of interest, it is not yet a widely recognized treatment for this dermatologic condition. This literature review serves to provide a consolidated overview of the current therapeutic implications of BTX‐A treatment for melasma and explore its proposed mechanisms of action.

**Objective:**

This review aims to provide a comprehensive analysis of the current evidence base for the efficacy of BTX‐A treatment on melasma.

**Methods:**

To gain a comprehensive understanding on the current theories regarding BTX‐A treatment on melasma, a literature review was conducted on all the available information using PubMed. A combination of keywords was used to maximize the search results, including “botulinum toxin,” “melasma,” “melanogenesis,” “neurotoxin,” “cholinergic system,” “BTX‐A,” and “UV‐induced melasma.” The search was not restricted by date, allowing for the inclusion of articles offering historical context and those providing the most recent findings. Ninety‐eight articles were reviewed to provide a consolidated update on the effectiveness of botulinum toxin A in reducing the appearance of melasma and potential mechanisms of action involved in doing so.

**Conclusion:**

Melasma is a challenging dermatologic condition due to its chronicity and various intrinsic and extrinsic factors that influence its pathogenesis. While current treatment options for melasma include topical, oral, and light‐based therapies, recent studies suggest that BTX‐A may hold potential as a viable treatment modality for melasma. Despite the mechanism of action remaining unclear, it is hypothesized that BTX‐A inhibition of Ach receptors on melanocytes could play a role in the reduction of melanogenesis. BTX‐A treatment in melasma‐affected skin demonstrates statistically significant reduction in hyperpigmented lesions associated with melasma.

## 1 Melasma

Melasma is a chronic dermatological condition that presents itself as brown or gray–brown hyperpigmented patches typically on photo‐exposed areas [1]. It affects approximately 5–6 million people in the United States alone, and 1% of the global population [2]. Melasma affects all races, however individuals of Hispanic, Latin American, Middle Eastern, Asia, and African origin (Fitzpatrick skin phototypes III–V) are more likely to be affected by melasma [3]. Melasma also predominantly affects women more than men, with the average age of onset being 30 years of age [1]. Melasma is classified by hypermelanosis, and traditional treatments focus on lightening the pigmentation and protecting the skin from sun exposure [3]. It is a multifactorial condition influenced by genetic, hormonal, and environmental factors. Despite its benign nature, melasma can significantly affect individuals' psychological well‐being due to its cosmetic implications and its difficulty to treat. Other forms of skin pigmentation such as postinflammatory hyperpigmentation (PIH) typically fade spontaneously over time after stimulus cessation, whereas melasma is a bit more complex in nature [4]. Melasma evolves from changes to several cutaneous layers to hyperactive melanocytes, which produce and transfer mature melanosomes to the whole epidermis [4].

Clinical presentation of melasma is characterized by light or dark brownish confluent macules that have irregular and sharply demarcated borders with a “stuck on” appearance [3]. The type of hypermelanosis in melasma patches can be characterized as epidermal (brownish), dermal (bluish gray), or mixed (brownish gray) [5]. Examination under a Wood's lamp allows clinicians to evaluate the clinical status of melasma, which can be broken down into the following four subcategories: the epidermal type (increased melanin in suprabasal, basal, and stratum corneum layers and the pigmentary lesions are emphasized under Wood's lamp), the dermal type (does not show enhancement under Wood's lamp, melanophages present in deep and superficial dermis), the mixed type (dermal and epidermal pigment type that shows no or slight enhancement with the Wood's light), and the inapparent type (specific to darker skin types, making it harder to notice at first glance) [6]. Under dermoscopy, melasma appears as pigmented dots, globules, more prominent vascularity, and telangiectasia [6, 7].

The diagnosis of melasma consists of symmetric reticulated hypermelanosis in three predominant facial patterns: centrofacial, malar, and mandibular [8]. The major clinical pattern in 50%–80% of patients is the centrofacial pattern which affects the forehead, nose, and upper lip while excluding the philtrum, cheeks, and chin [9, 10]. The malar pattern is contained to the malar cheeks on the face, while mandibular melasma is present on the jawline and chin. The mandibular pattern is thought to be common in older individuals and may be more related to severe photodamage [11]. A newer pattern termed “extra‐facial melasma” can also occur, which is located on nonfacial body parts, including the neck, sternum, forearms, and upper extremities [12]. Melasma can present variably, so researchers have developed standardized tools to enhance the ability to assess melasma severity and therapeutic efficacy of different melasma treatments in clinical trials [1]. The Melasma Area and Severity Index (MASI) is a scale that measures the extent of facial hyperpigmentation with a numeric score based on a calculation of the area of affected skin on the forehead, chin, and right and left malar cheeks [13]. The modified MASI (mMASI) has since been developed as a more reliable measure that correlates to the Melasma Severity Score, which is a global score including both objective data and patient's subjective evaluation [14].

## 2 Pathogenesis

The pathogenesis of melasma is complex, multifactorial, and not fully understood. Melasma is believed to arise from a combination of UV radiation, hormonal shifts (i.e., pregnancy, perimenopause, menstruation, sex hormones), skin inflammation (i.e., dermatitis, cosmetic procedures), as well as genetic predisposition [15]. Key biochemical players include melanocyte‐stimulating hormones (MSH), corticotropin‐releasing hormones, beta endorphin, tyrosinase, tyrosinase‐related protein 1 (Tyr‐p1), dopachrome tautomerase, and melanocyte‐stimulating receptors (MSRs).

### 2.1 UV Radiation

Solar radiation, specifically UVB, is the predominant environmental trigger of melasma. UVB and visible light stimulate keratinocytes, fibroblasts, and endothelial cells to secrete melanogenesis factors which stimulate melanocytes to produce melanin.

Several photobiological pathways are activated as soon as one is exposed to the sun, which ultimately lead to the upregulation of melanin‐stimulating receptors (MSRs). MSRs are located on the surface of melanocytes and stimulate the production and release of melanin once they are activated by MSHs [16]. MSRs also play a role in distributing melanin within the melanocyte and transfer melanin to keratinocytes which leads to visible coloration of the skin once the melanin accumulates there [16]. After UVB radiation exposure, melanogenesis is stimulated by keratinocytes and fibroblasts [1]. One primary pathway involves the secretion of stem cell factor (SCF), which can increase the proliferation of melanocytes [17]. Increased expression of SCF and melanogenesis‐related genes have been seen in the dermis of melasma‐affected lesions, as well as elevated mRNA levels of melanogenesis‐associated genes in these lesions [17]. Vascular endothelial growth factor (VEGF), produced by keratinocytes following UV damage, can also sustain melanocytes, contributing to the enhanced activity of melanocytes in melasma [18]. VEGF elevation has also been hypothesized to contribute to the increased vascularity found in melasma‐affected skin [19] Overexpression of nitric oxide, a powerful vasodilator that has been detected at the dermal–epidermal junction, is also believed to stimulate tyrosinase activity and enhance vascularity in melasma‐affected skin [16]. UVB radiation exposure can also cause an increase in reactive oxygen species, promoting melanogenesis [20]. Oxidative stress markers have been studied and found to be higher in melasma patients when compared to healthy individuals [21].

### 2.2 Genetics

An individual's family history can significantly impact their likelihood of developing melasma. Studies have shown that 55%–64% of patients affected by melasma have a positive family history, though no comprehensive genetic studies have been conducted [22, 23, 24]. Studies have also demonstrated that Fitzpatrick skin types II and III are less likely to have positive family histories for melasma than patients with darker skin types IV‐VI [25, 26]. In a global survey of 342 women, 48% of the individuals who have melasma have a positive family history, 97% of which were first degree relatives [1]. While there have not yet been any genome‐wide studies that relate specific genes to potentially causing melasma, transcriptional profiling has identified 279 genes that are expressed differently in lesional and perilesional skin [27].

### 2.3 Hormones

Hormonal influences also play a significant role in the pathogenesis of melasma, as evidenced by the higher prevalence of melasma during pregnancy, taking oral contraceptives or hormone replacement therapies, and menstruation [9, 10]. Particularly in the third trimester of pregnancy, women have elevated levels of pituitary, ovarian, and placental hormones, which can be a trigger for melanogenesis [28]. Sex hormones such as estrogen, specifically 17β‐estradiol (E2), and progesterone have been identified as factors involved in the regulation of pigmentation through nonclassical membrane‐bound receptors [29]. The development of hyperpigmentation during pregnancy may be associated with increased MSH, estrogen, and progesterone, which may result in increased transcription of tyrosinase and dopachrome tautomerase [30]. A cross‐sectional study of 400 pregnant women in Tehran showed that the prevalence of melasma was 15.8% [15]. In a multicentric cross‐sectional study in India, 15.2% of the subjects reported onset of melasma during pregnancy [31]. Alternatively, Hassan et al. studied melasma incidence in 36 menstruating women with melasma by comparing FSH, LH, prolactin, estrogen, and progesterone with controls of the same age [32]. There were higher levels of 17β‐estradiol at the beginning of the menstrual cycle among the melasma‐affected group than the control, suggesting that circulating estrogens may potentially be a risk factor and a “maintainer” of the disease [32]. In another study conducted in Pakistan with 138 women, the authors performed serum measurements of estradiol, progesterone, and prolactin [33]. The results showed a significant increase in estradiol levels both in the follicular and in the luteal phases in patients with melasma when compared to the controls [33]. As women reach menopause or surpass the age of 50, they experience a reduction in the number and activity of melanocytes [34]. Studies have shown a significant reduction in the prevalence of melasma after age 50 for this reason [34, 35].

Oral contraceptive pills (OCP) and hormone replacement have also been studied in regard to the pathogenesis of melasma when considering hormonal influences. It has been reported that 8%–34% of the women taking oral contraceptives or hormone replacement therapy (HRT) develop melasma [5]. Immunohistochemical studies have found an increased expression of progesterone and estrogen receptors in the epidermis and dermis of melasma‐affected skin, respectively [36]. As the female sex hormones that exist within OCP show to be critical for melasma development, the same association could be expected in postmenopausal females on hormone replacement therapy (HRT). Indeed, there are some case reports for melasma present in the postmenopausal phase [28]. Melasma in the forearms appears to be a comparatively common sign particularly in old age individuals and postmenopausal females using estrogen therapy supplementations [28]. Locci‐Molina et al. reported spontaneous improvements in patients with melasma after switching from a combined oral contraceptive to a hormone‐releasing intrauterine device, further emphasizing the role that longer acting systemic hormones present in OCP play in maintaining melasma [37] (Table 1).

**TABLE 1:** Factors contributing to melasma pathogenesis and each corresponding mechanism of pathogenesis.

Factor	Mechanism of pathogenesis	Reference(s)
UVB radiation	Keratinocyte stimulation → increased melanocyte production	Ogbechie‐Godec et al. [1] Khanna et al. [16] Kang et al. [17] Kim et al. [18] Griffiths et al. [64] Achar et al. [93]
Vascular	Growth factor induced angiogenesis and vasodilation	Kim et al. [19] Atefi et al. [94]
Endocrine	↑ Estrogen = ↑ melanogenesis	Handel et al. [5] Guinot et al. [9] Tamega et al. [10] Espósito et al. [15] Abdalla [28] Natale et al. [29] Goandal et al. [30] Sarkar [31] Hassan [32] Mahmood [33] Miot [34] Videira [35] Jang et al. [36] Locci‐Molina [37] Cohen et al. [95]
Genetic predisposition	↑ Family history = ↑ melasma diagnosis (mechanism unclear)	Ogbechie‐Godec et al. [1] Moin et al. [22] Adalatkhah et al. [23] Handel et al. [24] Ortonne et al. [25] Hexsel et al. [26] Kang et al. [27]
Inflammatory	↑ Skin inflammation = ↑ SCF in the melasma dermis = ↑ melanogenesis	Liu et al. [46]
cAMP accumulation	↑ Downstream melanin synthesis pathways	Lehraiki et al. [96]
Nitric Oxide	↑ Tyrosinase activity = ↑ melanogenesis	Khanna et al. [16] Jo et al. [20] Ennes et al. [57] Xiang et al. [75] Sarkar et al. [76] Baliña et al. [97] Nordlund et al. [98] Matsui et al. [99]
Skin barrier	↓ Lipid metabolism‐associated genes	Kang et al. [27]

## 3 Mechanism of Action

While the exact mechanism of melasma is still not fully understood, several key factors contribute to its pathophysiology. These include the complex crosstalk between melanocytes, keratinocytes, mast cells, fibroblasts, dermal vasculature, and endothelial cells [38, 39]. Melanin is a key component in the pigmentation of human skin, and its synthesis occurs in melanocytes once they are triggered by α‐melanocyte‐stimulating hormone (α‐MSH) as well as adrenocorticotropic hormone activation of the melanocortin‐1 receptor (MC1R) [40]. Once melanin is produced, it is transferred to epidermal keratinocytes, and eventually the overproduction and accumulation of melanin in keratinocytes can lead to pigmentary disorders such as melasma [41]. This process can be upregulated by several factors, especially UV–light exposure. UVB radiation can trigger keratinocytes to release α‐MSH, corticotropin, and lipid peroxidation, all of which upregulate melanogenesis through various pathways that involve cAMP, protein kinase A (PKA), cAMP response element‐binding protein (CREB), and microphthalmia‐associated transcription factor (MITF) activity [42]. UVB radiation also stimulates melanocyte‐specific genes, including tyrosinase, tyrosinase‐related protein 1 (Tyrp1), and dopachrome tautomerase, as well as the release of other key components to melanogenesis including corticotropin‐releasing hormone, proopiomelanocortin, melanocortin 1 receptor (MC1R), MC2R, and β‐endorphin [43, 44]. Prolonged exposure to UVB radiation can cause upregulation of SCF release from keratinocytes and fibroblasts which binds to the tyrosine kinase receptor c‐kit and causes activation of melanocytes [45]. This will also contribute to an increase in melanogenesis.

Inflammatory pathways in the skin can also contribute to the development or exacerbation of melasma. Prolonged UVB exposure can cause skin inflammation and fibroblast activation, which upregulates SCF in the dermis and increases melanogenesis [46]. Another hypothesized mechanism focuses on vascular abnormalities in cutaneous blood vessels [46, 47]. Studies have shown that vasodilation and aberrant angiogenesis may impact microcirculation in the skin, resulting in increased melanocyte activity and hyperpigmentation in the skin [47]. While these mechanisms have been hypothesized, the data for the true mechanism of action of melasma is limited, and not fully understood.

## 4 Current Treatments

Current treatments in the management of melasma focus on inhibiting the proliferation of melanocytes, formation of melanosomes, and advancement in their degradation [48]. Topical, oral, and procedural interventions achieve this by inhibiting melanin synthesis and melanocyte activity, removing melanin, and disrupting the melanin granules contained within melanosomes [49]. One of the first lines of therapies for melasma are topical treatments and photoprotection. UV and visible light avoidance can help reduce melasma flares. While iron oxide formulations in sunscreens block out visible and ultraviolet light and can be useful in managing melasma, some studies have shown that sunscreen alone cannot reduce a patient's risk of having a melasma relapse [50]. In repeated randomized controlled studies with 40–60 subjects, a comparison of broad‐spectrum UVA and UVB filters combined with visible light blockers, such as iron oxide, versus broad‐spectrum UV filters alone showed reduced melasma relapses in the former group [50, 51, 52].

Hydroquinone has historically been one of the most studied topical treatments for melasma and has been shown to lead to statistically significant melasma improvement [53]. Hydroquinone, also known as 1,4 dihydroxy benzene, works by inhibiting tyrosinase, which ultimately prevents the conversion of DOPA to melanin [54]. It is also believed to modify the formation of melanosomes, promote their breakdown, destroy melanocytes, and obstruct RNA and DNA synthesis [59]. It is generally applied topically as a cream, either on its own or in a combination treatment cream, ranging from 2% to 5% concentrations [48]. Noticeable pigment reduction occurs in a dose‐dependent manner, typically within 5–7 weeks, but the treatment must be sustained for at least 3 months to 1 year [55, 56]. Hydroquinone 4% has been found to fully or partially clear melasma in 95% of patients compared to 67% in the placebo group over a 12‐week period [57]. A twice‐daily application of 4% hydroquinone outperformed a placebo and improved melasma in 38% and 77% of patients, compared to 10% and 67% in placebo groups in two separate studies [57, 58].

Other nonhydroquinone‐based therapies for melasma include azelaic acid, kojic acid, tretinoin, ascorbic acid, arbutin, alpha hydroxy acids, niacinamide, and cysteamine [48]. Arbutin, azelaic acid, and kojic acid are tyrosinase inhibitors that cause cytotoxic effects on hyperactive melanocytes while having a minimal effect on normally pigmented skin [48]. Retinoids work by inhibiting tyrosinase transcription and its related proteins TRP‐1 and TRP‐2 which interrupt melanin synthesis after UVB exposure [53, 59, 60, 61, 62, 63], while also increasing keratinocyte turnover ultimately decreasing melanosome transfer [62]. Studies have shown topical tretinoin improves melasma by 68% in 38 patients over a 40‐week treatment period [64]. Alpha hydroxy acids work similarly to retinoids in accelerating epithelial cell turnover [48]. Ascorbic acid reduces dopaquinone to DOPA and acts as an antioxidant in addition to its photoprotective effect because it prevents absorption of ultraviolet radiation and promotes collagen synthesis [65]. It also inhibits melanogenesis by inhibiting tyrosinase activity via interacting with copper [65].

Cysteamine is a metabolite of the amino acid cysteine and inhibits melanin synthesis by disrupting the conversion of tyrosine to melanin in melanogenesis [66]. Interrupting tyrosinase activity reduces the production of melanin and can thus lighten areas of the skin affected by hyperpigmentation [66]. Cysteamine is available in topical creams as a depigmenting agent for melasma management [66]. In a formulation containing 5% cysteamine, a meta‐analysis of 120 patients showed a statistically significant improvement in melasma with a low probability of adverse effects [67]. Comparison studies have demonstrated that cysteamine 5% therapy is comparable to hydroquinone 4% in melasma management as shown by a statistically significant reduction in mMASI score and melanin index in a study of 60 patients at 2 months and 4 months posttreatment [68].

Topical corticosteroids, including hydrocortisone 0.1%, mometasone 0.1%, fluticasone 0.1%, and betamethasone valerate and fluocinolone acetonide 0.01%, are also used in the melasma management as they can prevent pigmentation from forming by nonselective suppression of melanogenesis and by acting as an anti‐inflammatory agent [69]. This therapy option is less common due to its adverse effects such as skin atrophy, facial hypertrichosis, acneiform eruptions, rosacea, and perioral dermatitis [49]. However, corticosteroids are typically used in a combination cream as opposed to a monotherapy as these creams often yield a high efficacy in the reduction of melasma [70]. The combination of hydroquinone 4%, tretinoin 0.05%, and fluocinolone acetonide 0.01%, known as the brand name Triluma, is the only US FDA‐approved treatment for melasma and is considered to be the gold standard treatment due to its demonstrated efficacy across all ethnicities [70].

Oral and injectable tranexamic acid is another effective treatment modality for melasma [48]. The exact mechanism for tranexamic acid on melasma remains unclear, however, a few studies have demonstrated that the inhibition of the plasminogen/plasmin pathway contributes to tranexamic acid‐induced reduction of melasma pigmentation [48, 71, 72]. Oral doses of tranexamic acid vary between 500 mg/day and 2.5 g/day for up to 6 months [48]. Studies have shown statistically significant improvements with oral tranexamic acid. One cross‐sectional study demonstrated that taking tranexamic acid 500 mg twice daily resulted in an early reduction in mean MASI score at 8 weeks onward compared with 250 mg once daily [72]. Tranexamic acid can also be injected directly into the affected skin to treat melasma in a 4 mg/mL solution [48]. Lee et al. demonstrated a reduction in patient MASI score in 8–12 weeks from baseline in 100 patients treated with intralesional tranexamic acid [73]. Interestingly, the efficacy of intralesional tranexamic acid was comparable to oral tranexamic acid, potentially implying that tranexamic acid is effective in treating melasma regardless of its vehicle [74].

While these topical and oral therapies continue to deliver effective results in treating and preventing recurrences in melasma, there are nonmedical treatment options including chemical peels, lasers, and light therapies that offer promise in the management of melasma. Chemical peels have demonstrated significant improvement in melasma reduction, but must be used with caution as they can cause irritation and postinflammatory hyperpigmentation especially in patients with Fitzpatrick skin phototypes III‐V [75]. Glycolic acid, salicylic acid, trichloroacetic acid, Jessner's solution, and phytic acid are all used to improve hyperpigmentation by removing unwanted melanin [76]. Laser therapy options include Q‐switched Nd‐YAG, Erbium: YAG, Q‐switched ruby, pulsed dye laser, fractional lasers, intense pulsed light (IPL), and radiofrequency microneedling. These can be used as monotherapy or in a multimodality approach especially in treatment‐resistant melasma cases [48]. Picosecond lasers at 1064 nm have also been shown to be an effective treatment for melasma.

## 5 Botulinum Toxin

Neurotoxins are a class of substances that harm the structure and functionality of the nervous system. Neurotoxins act by interfering with the body's communication system and disrupt the signaling between neurons. This interference typically occurs at the synapse, which is the contact point where neurons communicate with each other and other target cells [77]. Botulinum toxin (BXT) is a superfamily of neurotoxins that can block the release of acetylcholine and many other neurotransmitters from presynaptic vesicles by cleavage of specific proteins of the SNARE [78]. BXT is produced by Gram‐positive, rod‐shaped, spore‐forming anaerobic bacterium *Clostridium botulinum* [77]. This bacterium comes in several strains, including the A, B, C1, C2, D, E, F, and G serotypes; however, only toxins A and B are used clinically [77].

Initially, BTX was used to treat medical conditions such as blepharospasm, spasticity, cervical dystonia, and blepharospasm [77]. In 2002, a type of BXT, onabotulinum toxin A, was approved by the FDA for the treatment of glabellar rhytids. Since then, its cosmetic indications have expanded and several other botulinum toxins have been developed and FDA approved, as seen in Table 2 [79, 80, 81].

**TABLE 2:** FDA‐approved botulinum toxin formulations in the United States. US available neuromodulators.

Neurotoxin	Manufacturer	Year of FDA approval	Serotype	Onset of therapeutic effect	Duration of therapeutic effect	FDA‐cleared indications
Botox (onabotulinum toxin A)	Allergan Inc. Irvine, CA, USA	1989	A	3–14 days	3–6 months	Strabismus and blepharospasm, cervical dystonia, glabellar lines, axillary hyperhidrosis, chronic migraines and upper lip spasticity, urine incontinence, lateral canthal lines (Crow's feet)
Dyspor (abobotulinum toxin A)	Ispen Limited, Berkshire, UK	2009	A	24 h	3–6 months	Glabellar lines, cervical dystonia, upper limb spasticity in adults, lower limb spasticity in 2 + years old
Myobloc (rimabotulinum toxin B)	Solstice Neurosciences Inc. San Francisco, CA, USA	2009	B	3–14 days	3–6 months	Cervical dystonia, chronic sialorrhea
Xeomin (incobotulinum toxin A)	Merz Pharmaceuticals GmbH, Frankfurt am Main, Germany	2010	A	5–7 days	3–6 months	Glabellar lines, cervical dystonia, chronic sialorrhea, upper limb spasticity, blepharospasm
Jeuveau (prabotulinum toxin A)	Daewoong Pharmaceuticals, South Korea	2019	A	3–5 days	3–6 months	Glabellar lines
Daxxify (daxibotulinum toxin A‐lanm)	Revance Therapeutics Inc. Newark, CA, USA	2023	A	3–14 days	6 months	Glabellar lines, cervical dystonia
Letybo (letibotulinum toxin A‐wlbg)	Hugel Aesthetics Inc. South Korea	2024	A	3–5 days	3–4 months	Glabellar lines

## 6 Mechanism of Action

The mechanism of action of botulinum toxin consists of four steps. The toxin first binds to specific receptors on the surface of presynaptic cells, which is mediated by the C terminal of the heavy chain [77]. This initial step takes about 30 min to occur. The toxin is then internalized through energy‐dependent receptor‐mediated endocytosis [77]. In this step, the nerve cell's plasma membrane invaginates around the toxin receptor, forming a vesicle that encloses the toxin within the nerve terminal. Once this happens, the translocation process concludes [77]. After internalization, the disulfide bond is cleaved and the toxin's 50 kDa disulfide light chain is discharged across the endosomal membrane of the endocytic vesicle into the nerve terminal's cytoplasm [77]. In the last step, the light chain of serotypes A and E inhibit the release of acetylcholine by severing the cytoplasmic protein (SNAP‐25) necessary for docking acetylcholine vesicles on the nerve membrane's inner side within the nerve terminal [77]. This process of acetylcholine inhibition is temporary and causes muscle relaxation. When BXT is injected into the muscles of facial expression, these muscles that are attached to soft tissues and pull across the skin to allow us to use our expressions are now temporarily paralyzed [82]. The formation of facial wrinkles due to muscle contractions of the face will therefore also be reduced [82]. Clinically, the effects of botulinum toxin will onset within the first 4 days following injection, with the maximum effect occurring 1–4 weeks after injection [83]. BTX‐A does not cause permanent changes to nerve terminals in the targeted muscles and can last 3–4 months in the average patient [83].

## 7 Non‐Neuronal Cholinergic System of the Skin

The cholinergic system is composed of cholinergic neurons that release ACh, which then binds to nicotinic (n‐) and muscarinic (mAChRs) receptors to mediate transmission [84]. However, cholinergic communication has existed in the beginning of life among primitive organisms including algae, bacteria, fungi, and protozoa without the involvement of neurons [84]. Additionally, all of the components of the cholinergic system are present in mammalian non‐neuronal cells, including ACh, n‐, mAChRs, esterase, and high‐affinity choline uptake [84]. There is also evidence of ACh being synthesized outside of the nervous system, such as in epithelial, endothelial, and embryonic stem cells [84]. Due to these findings, the traditional idea of ACh functioning as solely a neurotransmitter needs to be revisited. The non‐neuronal cholinergic system (NNCS) of the skin refers to the presence and activity of cholinergic components in the skin that are not directly associated with neuronal signaling [85]. The term “cholinergic” refers to the neurotransmitter acetylcholine, and this type of signaling plays a significant role in various processes, including skin function and homeostasis [85].

The non‐neuronal cholinergic system is characterized by its ability to synthesize and release ACh [85]. In the skin, keratinocytes are the most significant cell types that can synthesize ACh in high concentrations [85]. These cell types also express both muscarinic and nicotinic receptors, the essential transporter proteins for auto‐/paracrine cholinergic loop, and the degradation enzyme AChE [85]. While keratinocytes are the main component of the non‐neuronal cholinergic system in the skin, fibroblasts and melanocytes also contain some molecular components of the NNCS as well [85]. The function of the NNCS in the skin is hypothesized to regulate the connection of keratinocytes, apoptosis, differentiation, proliferation, adhesion, and migration [86]. The effect of inflammation on the NNCS is complex. It is hypothesized that chronic inflammation causes an upregulation of ACh synthesis [85]. This was demonstrated in a study by Wessler et al. which showed a substantial increase of Ach levels in chronically inflamed atopic skin [87].

Melanocytes, the pigment‐producing cells of the skin, express ACh receptors, indicating that cholinergic signaling may influence melanin production and pigment regulation [85]. By interacting with these receptors, ACh can modulate melanocyte function and potentially impact melanogenesis. In melasma, dysregulation of melanin production leads to the development of hyperpigmented patches on the skin. While the specific role of ACh in the development or exacerbation of melasma is not fully understood, changes in cholinergic signaling and ACh levels may influence melanocyte function and melanogenesis, which could potentially contribute to the formation of hyperpigmentation disorders.

The non‐neuronal cholinergic system in the skin has been linked to the mechanisms of action of BTX‐A, particularly in the context of cosmetic applications. When BTX‐A is injected into the skin, it primarily acts on the neuromuscular junction to block the release of ACh [77]. By inhibiting ACh release, BTX‐A temporarily paralyzes the targeted muscles, leading to muscle relaxation and a reduction in dynamic wrinkles [77]. It is hypothesized that the effects of BTX‐A on acetylcholine release and cholinergic signaling could potentially impact skin functions beyond muscle activity [77]. Melanocytes contain ACh receptors on their surface, and when ACh release is upregulated, melanogenesis is also upregulated. If BTX‐A is blocking the release of ACh, this could potentially impact the function of melanocytes in producing pigment. In conditions like melasma, where there is an overproduction of melanin due to the nature of overactive melanocytes, this may offer a nontraditional therapeutic option for treating unwanted pigment.

## 8 Proposed Mechanism of Action in the Treatment of Melasma

The involvement of acetylcholine inhibition has led recent research to explore the potential of BTX‐A in the management of UVB induced hyperpigmentation, such as melasma. Although the direct relationship of ACh to melanogenesis remains unclear, ACh receptors have been shown to be located on the surface of melanocytes [88]. This finding suggests that ACh signaling may influence melanin production [88]. By blocking ACh release with BTX‐A, it is hypothesized that the interaction between ACh and melanocytes could be altered, potentially resulting in a reduction in melanin production and in the number of hyperpigmented patches in the skin that classify melasma [88]. Jung et al. supported this hypothesis with an in vitro and in vivo study using animal models to test the effect of BTX‐A on melanogenesis and other pathways [89]. In vitro, it was observed that following BTX‐A treatment, melanocyte dendricity and melanin contents were decreased significantly (*p* < 0.05) [89]. In vivo, BTX‐A demonstrated a statistically significant reduction in skin pigmentation, number of dihydroxyphenylalanine‐positive melanocytes, tyrosinase activity, melanin content, basic fibroblast growth factor, interleukin‐1 alpha, and prostaglandin E2 levels (all *p*s < 0.05) [89].

## 9 Use of BTX in the Treatment of Melasma

Suksantilap et al. tested BTX‐A on bilateral malar cheek melasma in 12 subjects [90]. For the BTX‐A injection group, hemi‐modified melasma area and severity (H‐mMASI) score was reduced at 2, 4, 8, and 12 weeks compared to baseline, though not statistically significant (*p* = 0.447) [90]. The Mexameter Melanin Index (MMI) in the BTX‐A group also gradually declined over time at 2, 4, 8, and 12 weeks and significantly differed with the baseline in the BTX‐A group (*p* = 0.014) [90]. Overall, the BTX‐A group also demonstrated global patient's satisfaction that was statistically higher than the control group (*p* < 0.05) [90]. Further supporting these findings, Jurairattanaporn et al. conducted a study on UVB irradiation induced hyperpigmented squares on the abdomen [91]. Seven days after the UVB was administered, five intervention groups were randomly assigned: control, 0.9% normal saline injection, 12 units (1:2.5), 6 units (1:5), and 4 units (1:7.5) of onabotulinum toxin injections [91]. The lightness index (L*), hyperpigmentation improvement score rated by a blinded physician, and subject satisfaction scores were then collected at 14, 21, and 28 days after injection [91]. Of all the treatment groups, the BTX‐A (1:2.5)‐treated site had a lower L* and pigmentation improvement score at all time points; however, there were no statistically significant differences between the groups [91].

Another interesting finding was highlighted in Erdil et al.′s study in which 31 patients were injected with BTX‐A for facial wrinkles [92]. Following injection, Melanin index (MI) was significantly lower in the upper face and forehead than in the lower face [92]. The forehead is much more photo exposed than the lower face, however, these areas were shown to be lighter after BTX‐A treatment than the parts of the face that were exposed to less sun [92]. Although the mechanism is unclear, it is possible that BTX‐A may have a direct effect on melanocytes or may be influencing other factors involved in pigmentation pathways involved in melasma progression. Further research is needed to fully understand the effect of BTX‐A on skin pigmentation to determine the optimal therapeutic options for melasma management (Table 3).

**TABLE 3:** Current literature on botulinum toxin in the treatment of melasma‐affected skin, first author, reference number, type of study, and corresponding finding.

First author, year	Type of study	*n* (site)	Results
Erdil [92], 2023	Prospective	31 (forehead, glabella, crow's feet)	Melanin index (MI) was significantly lower in the upper face and forehead following botulinum toxin treatment.
Jurairattanaporn [91], 2022	Prospective	15 (abdomen)	The BTX‐A (1:2.5)‐treated site had a lower degree of hyperpigmentation at all time points, as measured by mean L* and hyperpigmentation improvement scores. However, there were no statistically significant differences between the groups. Participants were most satisfied with the control site.
Suksantilap [90], 2022	Prospective	12 (bilateral malar face)	Hemi‐modified melasma area and severity (H‐mMASI) score was reduced in the botulinum toxin type A injection group at 2, 4, 8, and 12 weeks compared to baseline, but there was no statistically significant difference (*p* = 0.447), Mexameter Melanin Index (MMI) gradually declined over time at 2, 4, 8, and 12 weeks and significantly differ with the baseline in the botulinum toxin type A injection group (*p* = 0.014), global patient's satisfaction was statistically higher than without treatment group (*p* < 0.05).
Jung [89], 2019	Experimental	In vitro (human epithelial cells) and In vivo (animal model)	In vitro: melanocyte dendricity and melanin contents were decreased significantly (*p* < 0.05) after botulinum toxin type A treatment. In vivo: botulinum toxin type A suppressed skin pigmentation, the number of dihydroxyphenylalanine‐positive melanocytes was significantly lower than in the control side, tyrosinase activity and melanin content were also significantly reduced following botulinum toxin treatment, botulinum toxin type A significantly reduced the amounts of basic fibroblast growth factor, interleukin‐1 alpha, and prostaglandin E2 (all *p* < 0.05).

## 10 Conclusion

Melasma presents a complex challenge in dermatology, characterized by its persistent nature and multifaceted etiology influenced by both intrinsic and extrinsic factors. A variety of treatment options, including topical agents, oral medications, and light‐based therapies are available for managing melasma. However, the search for continuous and efficacious therapies remains ongoing, with a focus on safety and effectiveness for long‐term management for all skin types. BTX‐A, known for its efficacy in treating various medical conditions and dynamic rhytids, has emerged as a potential therapeutic option for melasma. Recent evidence suggests an impact of BTX‐A on melanin production through its inhibition on acetylcholine receptors on melanocytes, offering promise for its role in melasma management. However, further well‐designed clinical trials and extended follow‐up studies are necessary to confirm its efficacy and safety profile in the treatment of melasma. Comparative research studies comparing BTX‐A with conventional therapies and combination treatments used in melasma management could provide valuable insights into the potential benefits of BTX‐A in a multimodality context. Investigating the mechanisms of action of BTX‐A on melanogenesis and pigmentation pathways may reveal novel targets for therapeutic intervention in melasma.

## 11 Conflicts of Interest

The authors declare no conflicts of interest.

## Data Availability

The data that support the findings of this study are available on request from the corresponding author. The data are not publicly available due to privacy or ethical restrictions.
